# Interest in Home Medical Care Among Middle-Aged and Older Residents Living in the Mountainous Areas of Sagamihara City

**DOI:** 10.7759/cureus.91352

**Published:** 2025-08-31

**Authors:** Takayuki Hoshiyama, Makoto Kaneko, Nana Sugimoto, Mika Nagashima, Satoshi Yamashita, Ayako Hoshiyama, Naoyoshi Aoyama

**Affiliations:** 1 Department of General Medicine, Kitasato University School of Medicine, Sagamihara, JPN; 2 Department of Health Data Science, Yokohama City University, Yokohama, JPN; 3 Department of General Medicine, Chigira Clinic, Sagamihara, JPN; 4 Department of General Medicine, Fujino Clinic, Sagamihara, JPN; 5 Department of General Medicine, Aonohara Clinic, Sagamihara, JPN

**Keywords:** aging population, cross-sectional survey, home healthcare interest, home medical care, rural healthcare, sagamihara city

## Abstract

Objective

In Japan, the aging population is rapidly increasing, with many older individuals preferring to spend their final days at home. However, despite ongoing policy efforts and efforts to promote home medical care, the transition from hospital to home remains slow, likely reflecting a mixture of system-level and patient-level factors. This study aimed to identify patient-related factors that influence awareness and interest in transitioning to home medical care using a questionnaire survey among residents aged 45 years and older in rural Sagamihara City.

Patients and methods

A cross-sectional survey was conducted at three rural clinics in Sagamihara City between January and December 2023. Factors influencing interest in home healthcare among participants aged 45 years and older, including age, self-rated health, travel time to clinics, hospitalization history, multiple clinic visits, household composition, and transportation methods, were examined. Statistical analyses utilized Cochran-Armitage trend tests for ordinal variables and Pearson's χ^²^ tests for categorical variables.

Results

Interest in home healthcare increased with age, reaching 51 of 69 respondents (73.9%) among those aged 85 years and older. In addition, 700 of 793 respondents (88.3%) indicated that home healthcare in their residential area needs improvement, and this percentage tended to increase with age. Lower self-perceived health and longer travel time to medical facilities were also associated with higher interest in home-based medical care. Women and those relying on family-driven private vehicles showed greater interest in home-based medical care. No statistically significant differences were observed for hospitalization history, multiple clinic visits, or household composition, although certain trends were noted.

Conclusion

Older age, lower self-perceived health, and longer hospital visit times were associated with greater interest in home medical care. Significant differences were also observed in sex and private car use. These findings may suggest a need to enhance home healthcare services tailored to patients' specific needs.

## Introduction

As of September 2024, Japan's older population reached 36.25 million (aging rate, 29.3%), and the hospital death rate remained high at 64.5% [1,2]. Under these circumstances, nearly half of the older individuals with incurable diseases express a preference for spending their final days at home [3,4]. This highlights the urgent need to promote home medical care by shifting the primary setting for end-of-life care from hospitals to the home [4]. However, according to the 2022 data from the Ministry of Health, Labour and Welfare, the home death rate was only 17.4%, which is much lower than the aforementioned hospital death rate [2]. Although home deaths have gradually increased in recent years, the proportion has remained largely unchanged since it dropped below 20% in 1995, following 1976 when hospital deaths first surpassed home deaths [5,6]. In response, the Ministry of Health, Labour and Welfare aims to establish a community-based integrated care system by 2025, actively promoting home-based medical care to ensure that those wishing to remain at home can do so [6,7]. However, compared to other developed countries, significant disparities persist. For example, the home death rates in the United States and the United Kingdom are 30% and 23%, respectively, reflecting an increase of approximately 30% since the mid-2000s [8,9].

The transition to home-based care is delayed due to the mutual influence of issues faced by both healthcare providers and patients, as well as their families. The shortage of physicians and nurses involved in home-based care, as well as inadequate infrastructure and support systems, directly impacts the decision-making of patients and their families regarding home-based medical care [10]. In situations where support from healthcare providers is lacking, patients and their families tend to feel more anxious about the burden of care on the family and their ability to respond to sudden health changes at home. Additionally, awareness of home-based medical care remains low. Consequently, even patients who are interested in home-based medical care find it challenging to determine the optimal timing for initiating such care [6,11].

Furthermore, the definition for the appropriate timing of introducing home-based medical care is not standardized, and decisions largely rely on the subjective medical judgments of physicians, nurses, and care managers [12,13]. Under these circumstances, healthcare providers must consider the living environments and values of patients adequately and provide them with timely information and support. This patient-centered approach is considered essential for facilitating the timely initiation of home-based care. However, limited studies have investigated the specific factors and timing affecting the introduction of home-based medical care, including the relationship between the challenges faced by healthcare providers and patient decision-making.

This study aimed to identify patient-related factors that influence awareness and interest in transitioning to home-based medical care, using a questionnaire survey among residents aged 45 years and older in rural Sagamihara City.

## Materials and methods

Research design

A cross-sectional survey was conducted from January 1, 2023 to December 31, 2023, including patients who visited three clinics in the rural mountainous areas of Sagamihara City, Kanagawa Prefecture: Aonohara Clinic, Chigira Clinic, and Fujino Clinic.

Setting

Sagamihara City is a core regional city of Japan with a population exceeding 700,000. We collected data from three clinics located in Tsukui, Sagamiko, and Fujino districts. These facilities are the only municipal, non-bed outpatient clinics operated by Sagamihara City, offering outpatient and home visit services, playing a key role in transitioning patients from outpatient to home healthcare. These clinics were selected because they are the only municipal outpatient facilities providing both ambulatory and home visit care in this mountainous region, and they are operated directly by the city. Their unique role and public status made them appropriate and accessible for surveying the local population. Therefore, a convenience sampling approach was adopted, focusing on these representative community-based clinics. The mountainous area spans 218 km², covering 66% of the city's total area, yet houses only 39,279 residents (5.4% of the total population) [14]. Moreover, 39.9% of residents are aged 65 years and older, exceeding the national average by over 10% as of 2023 [15]. These demographics suggest a rising demand for home healthcare in this region.

Patient inclusion

This study included all patients who visited the designated clinic during the study period and could voluntarily complete the questionnaire. The questionnaire is the part of a larger project, research on outpatients at three Sagamihara City clinics, which aimed to assess clinic operations; therefore, we did not establish specific exclusion criteria. Participants received verbal and written explanations of the survey, and informed consent was obtained using an opt-out method. To target age groups expected to have greater home healthcare needs due to aging, patients aged 45 years and older were included. This age range represents the period when the use of medical and caregiving services typically increases and interest in home healthcare is likely to grow.

**Figure 1:**
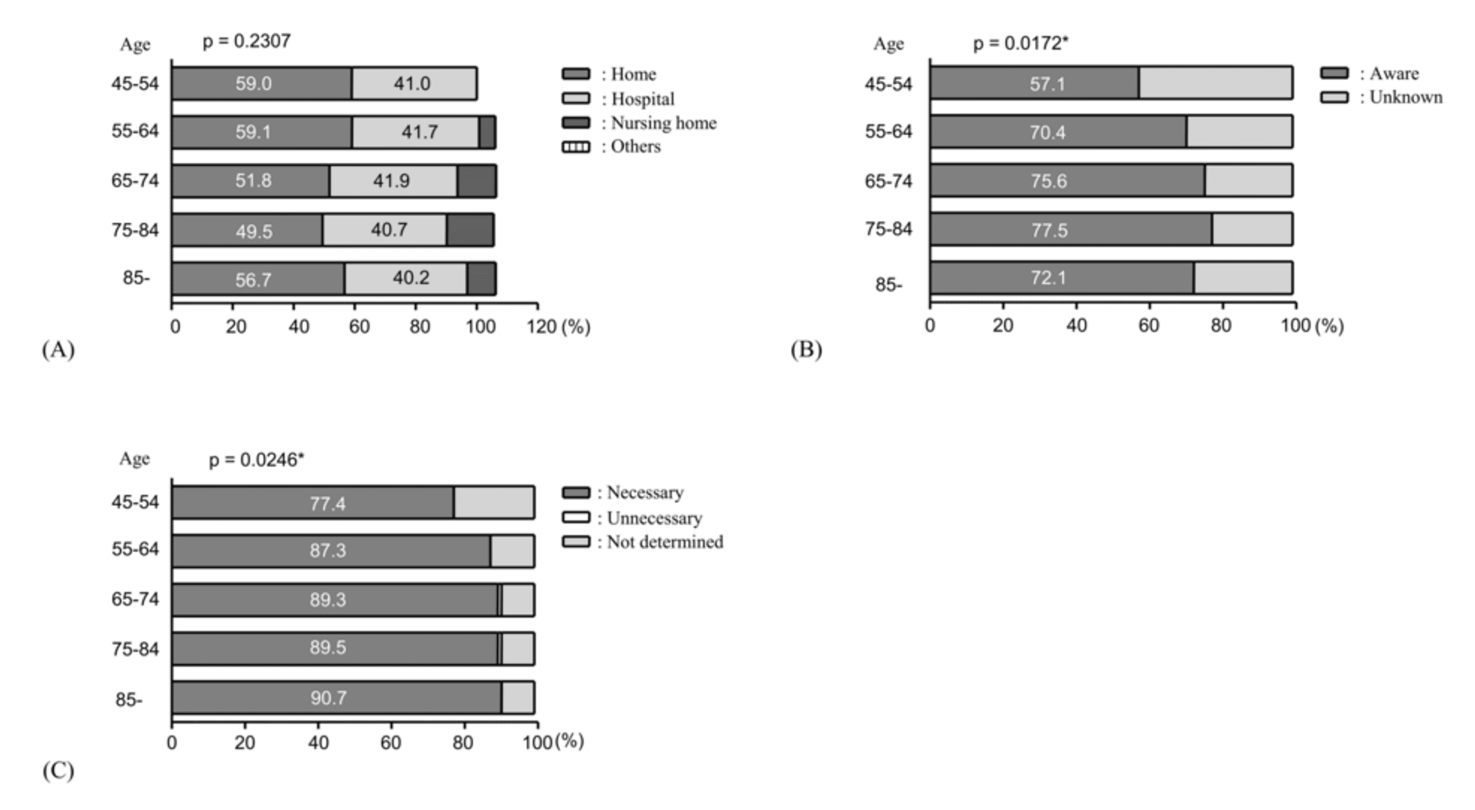
Age trends in basic knowledge and needs for home medical care. The Cochran-Armitage test for trend was used for the analysis. Statistical significance was defined as p<0.05, and significant p-values are denoted with an asterisk (*). (A) Preferences for long-term care locations. As respondents were allowed to select multiple options, the total percentage exceeds 100% (p=0.2307). (B) Knowledge of home medical care (*p=0.0172). (C) Need for more home medical care in one’s own residential area (*p=0.0246).

The questionnaire was distributed during the participants' initial visit during the study period and collected on-site. If participants were unable to complete it due to time constraints or other reasons, they were given another opportunity at their next visit. Details of the questionnaire content are provided in the Appendix (see Tables 3, 4). Some questionnaire items, specifically those corresponding to Figures 1B, 1C, were identical to items used in a citywide resident survey previously conducted by Sagamihara City, which targeted all residents in the municipality. This alignment allowed for comparability with existing local data. The remaining items were developed by the study team based on the study objectives and reviewed for face validity by clinicians familiar with the patient population; however, no formal pilot testing or statistical validation was performed. Details of these items are further discussed in the Discussion section. Although no formal exclusion criteria were established in advance, during this study, the attending physicians could refrain from administering the questionnaire if they judged that a patient was unable to respond accurately (e.g., suspected cognitive impairment). While this operational decision was not explicitly described in the original study protocol, it was implemented within the scope of the protocol approved by the ethics committees of Kitasato University School of Medicine and Sagamihara Red Cross Hospital.

Variables

The primary outcome was defined as “the interest in home healthcare,” which was assessed using the following response options: I am not considering using home healthcare at this time, I would consider it under certain conditions, I am actively considering it, and I am unsure at this time. Responses of “I would consider it under certain conditions” and “I am actively considering it” were categorized as currently interested in home healthcare.

While the primary outcome of this study-self-reported “interest in home medical care”-provides useful insight into patients' attitudes, it does not necessarily capture actual future utilization or behavioral intention in real-world settings. We selected this measure because it is a practical, low-burden way to assess attitudes in a large outpatient population and because more objective utilization data were not available within the scope of this study. Nevertheless, the measure serves as a proxy measure rather than a definitive predictor of actual uptake.

Various factors that could influence this outcome, including age, self-rated health, travel time to the clinic, hospitalization in the past year, concurrent visits to other medical institutions, household composition, and means of transportation, were collected and analyzed based on the questionnaire responses. Items marked as "not answered" or "unsure" were excluded from the analysis. Data aggregation and analysis were conducted to examine factors associated with the interest in home healthcare.

Secondary outcomes included preferred location for long-term care, awareness of home healthcare, and perceived need to enhance home medical care services in residential areas. The influence of age on these outcomes was analyzed using the questionnaire data in a similar manner. Data on other potential confounding factors, such as educational attainment, household income, and access to information and communication technologies, were not collected in this study due to feasibility constraints in the patient-administered questionnaire format.

Sample size

The sample size was determined in advance based on the primary outcome. Using G*Power software (Version 3.1; Heinrich-Heine-Universität Düsseldorf, Düsseldorf, Germany), calculations were performed with the following parameters: power of 0.8, significance level of 0.05, and effect size of 0.5. The results indicated that 67 participants per group (134 in total) were required.

Statistical analysis

Statistical analyses were performed using JMP Pro 17 software (SAS Institute Inc., Cary, NC, USA) and GraphPad Prism 5.02 software (GraphPad Software Inc., San Diego, CA, USA). Continuous variables are summarized as median and interquartile range (IQR), while categorical variables are reported as real counts and percentages. Statistical analyses included the Cochran-Armitage trend test to assess associations between age groups, self-rated health, travel time to the clinic, interest in home healthcare, and secondary outcomes (preferred location for long-term care, awareness of home healthcare, and perceived need for improvement in home healthcare services). Pearson's χ² test compared groups by sex, hospitalization in the past year, concurrent visits to other medical institutions, household composition, and means of transportation. The Bonferroni method was applied for multiple group comparisons. The results of chi-square analyses, including χ² values and p-values for each comparison, are summarized in a separate table. A P-value of less than 0.05 was considered statistically significant, and significant differences are indicated with an asterisk (*). For each analysis, listwise deletion was applied, excluding cases with missing or “unsure” responses for the variables of interest. The number of excluded cases for each variable is provided in the Appendix (see Table 4). To explore factors related to the interest in home medical care, logistic regression analysis was performed. Categorical variables were converted into dummy variables for the analysis. The reference categories were set as follows: age (45-54 years), subjective health perspective (healthy), time to hospital (less than five minutes), sex (male), and means of transport to hospital visits (private car (self-driven)). Logistic regression analysis was conducted using Python (version 3.11.12; Python Software Foundation, Wilmington, DE, USA) and the scikit-learn library (version 1.6.1; scikit-learn developers, Paris, France).

## Results

Response rate and participant characteristics

The valid response rates were as follows: Aonohara Clinic, 192 participants (30.5%); Chigira Clinic, 230 participants (55.6%); and Fujino Clinic, 385 participants (49.9%), totaling 807 participants with an overall response rate of 44.5%. Table 1 presents the characteristics of respondents. The median age at the three clinics was 72, 74, and 75 years, respectively. The median number of visits in the past year was 11, 12, and 12, and the median travel time to the clinics was five, five, and 10 minutes, respectively. None of these indices differed significantly across clinics. Additionally, all three clinics showed a slight predominance of female respondents.

**Table 1:** Characteristics of respondents from the three clinics. *Results are expressed as median (interquartile range).

	Aonohara Clinic (n=192)	Chigira Clinic (n=230)	Fujino Clinic (n=385)	Overall (n=807)
Age*	72 (63–78)	74 (62-81)	75 (68-82)	74 (65-81)
Age categories (years), n (%)				
45–54	21 (10.9)	26 (11.3)	16 (4.2)	63 (7.8)
55–64	40 (20.8)	41 (17.8)	52 (13.8)	134 (16.6)
65–74	54 (28.1)	57 (24.8)	116 (30.1)	227 (28.1)
75–84	63 (32.8)	78 (33.9)	140 (36.4)	281 (34.8)
≧84	14 (7.3)	28 (7.3)	60 (15.6)	102 (12.6)
Sex, n (%)				
Male	88 (45.8)	112 (48.7)	190 (49.4)	390 (48.3)
Female	104 (54.2)	118 (51.3)	195 (50.6)	417 (51.7)
Number of visits last year*	11 (6-12)	12 (9-13)	12 (10-12)	12 (9-13)
Visiting time*	5 (5-10)	5 (3-10)	10 (5-10)	5 (5-10)

Basic knowledge and needs regarding home medical care

The majority of respondents (418 (53.3%)) preferred home as their desired place of care for long-term medical needs. Cochran-Armitage trend tests revealed no statistically significant trends across age groups. However, the preference for home healthcare was the lowest among those aged 75-84 years (135 (49.5%)), which increased slightly in those aged 85 years or older (55 (56.7%)) (Figure 1A).

Regarding awareness of home medical care, 584 (73.5%) of the respondents reported familiarity with the concept. Cochran-Armitage test identified significant trends across age groups, with awareness rates exceeding 70% in groups aged 55 years and older (Figure 1B).

The overall number of respondents who answered “necessary” to whether home medical care should be enhanced in their residential area was 700 (88.3%). Statistically significant trends were observed across age groups, with particularly high percentages (approximately 90%) in groups aged 55 years and older (Figure 1C).

Trends in interest in home medical care

**Figure 2:**
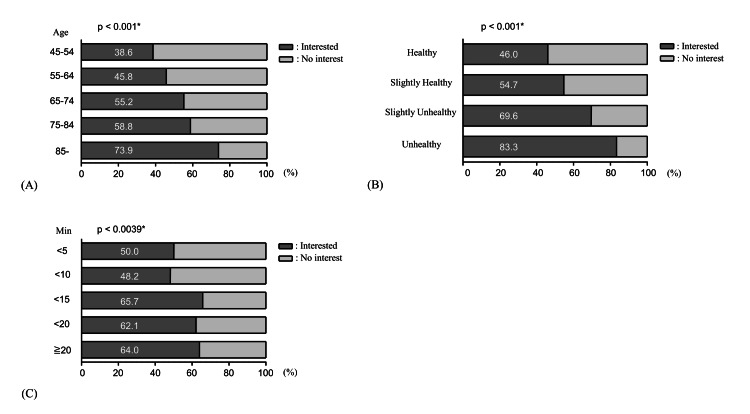
Trends in interest in home medical care. The Cochran-Armitage test for trend was used for the analysis. Statistical significance was defined as p<0.05, and significant p-values are denoted with an asterisk (*). (A) Age and interest in home medical care (*p<0.001). (B) Subjective health perspective and interest in home medical care (*p<0.001). (C) Time to hospital and interest in home medical care (*p=0.0039).

Figure 2 illustrates trends in interest in home medical care by various factors. An analysis by age group showed a statistically significant increase in interest with advancing age (Figure 2A). Among the youngest group (n=44), 17 (38.6%) expressed interest, compared to 51 (73.9%) of those aged 85 years or older (n=69). Participants were divided into two groups based on the age of 75 years. Among those younger than 75 years, 151 (49.8%) expressed interest, whereas 161 (62.9%) of those aged 75 years and older did so, indicating a notable difference. Furthermore, among those classified as “interested in home healthcare,” 42 (38.3%) of participants aged 75-84 responded “I am actively considering it,” the highest among all age groups. In contrast, the proportion ranged from 23.5% to 31.4% in other age groups.

The relationship between self-rated health and interest in home healthcare revealed a statistically significant trend: individuals who perceived their health as poor demonstrated greater interest in home healthcare (Figure 2B). While 238 (52.3%) of those identifying themselves as healthy or somewhat healthy expressed interest in home healthcare, this proportion increased to 74 (71.2%) among those who considered themselves unhealthy or somewhat unhealthy, indicating a difference of approximately 20 percentage points.

Interest in home medical care also correlated with travel time to the clinic. Respondents with longer travel times showed greater interest. Notably, 148 (48.7%) of respondents with a travel time of less than 10 min expressed interest, compared to 162 (64.5%) of those with a travel time of 10 min or more, indicating a significant difference (Figure 2C).

Interest in home medical care by patient status

**Figure 3:**
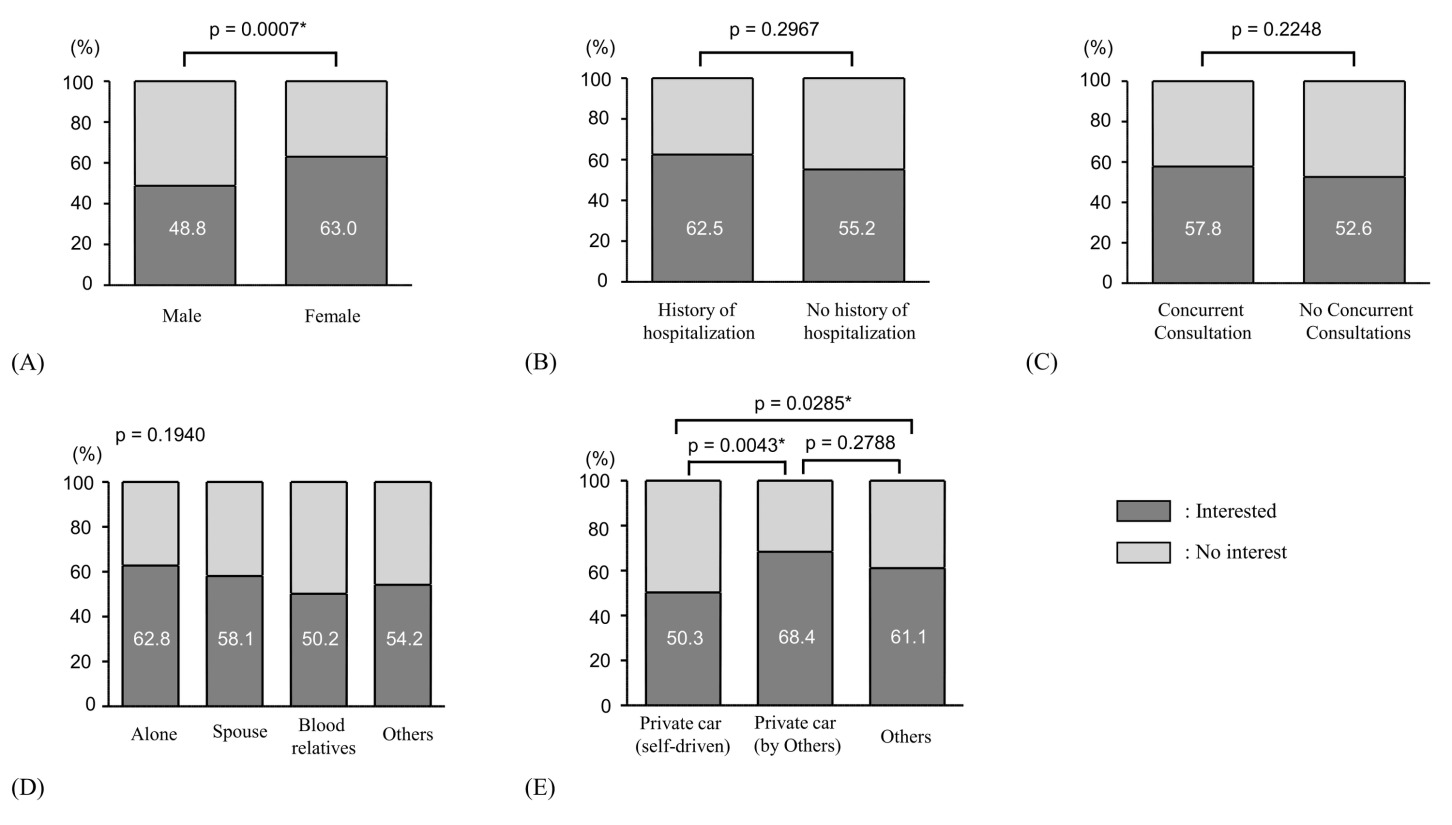
Interest in home medical care by patient status. Pearson's χ² test was applied for group comparisons. For multiple group comparisons, the Bonferroni method was applied for correction. A p-value of <0.05 was considered statistically significant and is denoted with an asterisk (*). Statistical details are presented in Table 2. (A) Gender and interest in home medical care. (B) Hospitalization in the past year and interest in home medical care. (C) Concurrent visits to other hospitals and interest in home medical care. (D) Household composition and interest in home medical care. (E) Means of hospital visits and interest in home medical care.

A comparison of interest in home healthcare by patient status is shown in Figure 3. Table 2 presents the statistical significance (χ² values and p-values) corresponding to each subgroup comparison shown in Figure 3.

Regarding sex differences, women demonstrated significantly higher interest levels than that demonstrated by men (Figure 3A). Among those classified as “interested in home healthcare,” the proportion of respondents who answered “I am actively considering it” was 43 (31.4%) for men (n=137) and 59 (33.7%) for women (n=175), with no significant difference observed.

While no statistically significant difference was observed based on hospitalization history in the past year, respondents with a hospitalization history showed numerically higher interest than that demonstrated by those without (hospitalized: 35 (62.5%), not hospitalized: 276 (55.2%)), although the difference was not statistically significant (Figure 3B).

Similarly, no statistically significant difference was found regarding concurrent visits to other hospitals, although respondents receiving care at multiple facilities had numerically higher interest levels (189 (57.8%)) than those had by respondents without concurrent care (121 (52.6%)); however, the difference was not statistically significant (Figure 3C). Regarding the history of hospitalization within the past year and outpatient visits to other hospitals, no significant differences were found, even when only those who responded “I am actively considering it” were analyzed.

The analysis of interest by household composition categorized respondents into four groups: living alone, living with a spouse, living with a parent or child, and others. While no statistically significant differences were observed across groups (Figure 3D); numerically, respondents living alone had the highest interest in home medical care (54 (62.8%)), whereas those living with a parent or child showed the lowest interest (100 (50.3%)). Among the respondents who reported active interest in home healthcare, the proportion of those living with a parent or child was significantly lower than that of those living alone or with a spouse (26 (13.1%) vs. 23 (26.7%); p=0.006 and 26 (13.1%) vs. 47 (21.2%); p=0.029, respectively). Other pairwise comparisons did not reach statistical significance.

Interest in home medical care based on means of transportation was compared by classifying patients into three groups: private car (self-driven), private car (driven by family members or others), and other means (e.g., walking, motorcycle, or public transportation). A statistically significant difference in interest in home healthcare was observed by means of transportation. Specifically, a significant difference was identified between the private car (self-driven) and private car (family-driven) groups (p=0.0043), indicating notable variability in interest levels (Figure 3E).

**Table 2:** Results of Pearson's χ² tests for interest in home medical care by patient status. Corresponding to Figure 3. *p<0.05 was considered statistically significant and is indicated with an asterisk (*).

Comparison	χ² value	p-value
(A) Gender	11.418	0.0007*
(B) Hospitalization in the past year	1.089	0.2967
(C) Concurrent visits to other hospitals	1.473	0.2248
(D) Household composition	4.713	0.1940
(E) Means of hospital visits	10.647	0.0049*
Private car (self) vs. private car (other)	8.166	0.0043*
Private car (self) vs. others	4.799	0.0285*
Private car (others) vs. others	1.173	0.2788

Contribution to interest in home medical care

In the logistic regression analysis, being aged 85 years or older (odds ratio (OR): 3.70, 95% confidence interval (CI): 1.51-9.03, p=0.004), being female (OR: 1.67, 95% CI: 1.15-2.41, p=0.006), having a perception of being “somewhat unhealthy” (OR: 2.30, 95% CI: 1.26-4.22, p=0.006), and having a perception of being “unhealthy” (OR: 4.92, 95% CI: 1.00-24.05, p=0.05) were significantly associated with interest in home medical care (p ≤ 0.05). The odds ratio was highest for those who perceived themselves as “unhealthy,” followed by being aged 85 years or older, having a perception of being “somewhat unhealthy,” and being female. No significant associations were found for the other variables (p > 0.05).

## Discussion

This study examined patients' interest in home-based medical care and its associated factors. To aid in interpreting the findings, we drew on Andersen's Behavioral Model of Health Services Use -- which emphasizes the role of predisposing factors (e.g., age and sex), enabling resources (e.g., transportation and knowledge), and perceived need -- and the Health Belief Model, which focuses on perceived benefits and barriers influencing health-related behavior.

A common reason for individuals to consider is the need for long-term care due to illness or declining health. Previous nationwide surveys have reported that approximately half of respondents choose home in such circumstances [3,4,16]. In this study, 53.3% of participants expressed a preference for home, showing no significant deviation from prior findings.

When selecting a long-term care setting, the issue of burden can be broadly categorized into two major concerns: the physical and psychological burdens on both caregivers and care recipients, as well as the financial burden [13]. Home-based care may reduce medical costs but places significant daily responsibilities on family members, including managing health fluctuations. Care recipients often experience stress stemming from feelings of guilt for burdening their families [17,18].

As both individuals and their caregivers age, providing support becomes increasingly difficult, leading care recipients to worry more about imposing on family members. Consequently, preference for home-based care declines with age but rises again among individuals aged 85 years and older. This resurgence may stem from reduced concerns over caregiving burdens, as solitary living rates among individuals aged 75 years and older reached approximately 20% in this study, double that of those under 75 years (data available from the corresponding author upon reasonable request).

Awareness of home healthcare and its availability in the residential area were also key enabling factors. Awareness increased with age, rising sharply to over 70% among those aged 55 years and above. The percentage of those who think that home medical care in their residential district needs to be further enhanced was 88.3% overall and nearly 90% among those aged 55 years and above. These results closely align with findings from a local survey conducted in the same area [15]. As individuals age, they become more proactive in seeking information about future healthcare options and increasingly advocate for the enhancement of home-based medical care in their residential areas owing to personal concerns. However, younger age groups demonstrated lower awareness, perceiving home-based care primarily as a service for older individuals. Expanding education and information campaigns to younger generations may increase both awareness and interest.

Regarding age and interest in home healthcare, a trend was observed in which interest tended to increase with advancing age, beginning around the age of 65 years and peaking at 75-84 years. This may be related to retirement timing, the mailing of information about the “Medical Care System for the Elderly Aged 75 and Over,” and recognition of physical decline. For the oldest group (≥85 years), renewed interest may reflect reduced caregiving concerns due to solitary living. These findings suggest that older individuals may be more likely to consider home healthcare as a means to reduce the burden of outpatient visits and potentially maintain their quality of life despite physical limitations [19].

Individuals perceiving their health as unhealthy were more likely to show interest in home-based medical care. Among those who reported being healthy or somewhat healthy, 52.3% expressed interest, compared to 71.2% among those who considered themselves unhealthy or somewhat unhealthy, reflecting a difference of approximately 20%. Poor health may be associated with greater perceived benefits of home care, possibly due to frequent medical needs, mobility challenges, and the strain of visiting medical facilities.

Longer commuting times to medical appointments were associated with increased interest in home healthcare. While 48.7% of participants preferred home healthcare with commutes under 10 minutes, this increased to 64.5% for commutes over 10 minutes. Time and transportation burdens, especially for older adults or those in areas with poor transportation, may therefore drive demand for home healthcare services.

Women showed significantly stronger interest in home healthcare than men. This may be attributed to women's generally higher engagement with health and medical issues, as well as their greater likelihood of assuming caregiving roles, which could heighten their focus on home healthcare [20,21].

Respondents with a history of hospitalization were observed to have slightly greater interest in home-based care, although the difference was not statistically significant. Hospitalization experiences may increase awareness of home-based care's role in managing conditions and preventing readmissions. Patients visiting multiple clinics also showed slightly higher interest, possibly due to increased exposure to healthcare systems and alternative care options. While these trends were not significant, they may still reflect experiential factors influencing attitudes toward home-based care.

Interest in home-based care varied by household type but showed no statistically significant differences. Individuals living alone tended to have the highest interest (62.8%), while those living with parents or children tended to have the lowest interest (50.3%). Solitary living may heighten concerns about health management and increase interest in home-based care [22]. In the analysis limited to those reporting active interest, the proportion living with a parent or child was significantly lower than among those living alone or with a spouse, suggesting that the presence of close relatives may reduce perceived need.

Transportation mode showed a significant difference, particularly between self-driven and family/others-driven privately owned vehicles. Individuals who drive themselves may find commuting easier and be less likely to perceive going out as burdensome, resulting in lower reliance on home-based care. Conversely, those relying on others for transportation may anticipate difficulties if such support diminishes, increasing their consideration of home-based care. These findings suggest that transportation may be an important enabling factor in Andersen's model and a perceived barrier in the Health Belief Model, which could inform future service delivery and policy considerations.

The logistic regression analysis results suggest that self-perceived health status plays a particularly important role in contributing to an increased interest in home medical care. Older adults -- particularly those aged 85 years and above -- and being a woman were also associated with higher interest, which may inform the development of well-structured support systems and comprehensive information dissemination strategies. Some variables identified in the logistic regression analysis had wide confidence intervals and p-values near the conventional significance threshold, indicating lower precision of these estimates. These associations should therefore be interpreted with caution and considered exploratory, as further studies with more comprehensive covariate adjustment are needed (see Limitations). By focusing on a highly aged rural population and utilizing clinic-based data from patients currently receiving care, this study provides context-specific insights that help to confirm, contextualize, and extend existing findings. Such data are valuable for local policymakers and healthcare providers aiming to promote appropriate home-based care strategies in under-resourced and high-need communities.

Limitations

This study is based on a survey aimed at understanding the current situation. While no specific exclusion criteria were defined beforehand, individuals unable to complete the survey may have been effectively excluded, meaning their perspectives may not be reflected in the findings. This operational approach, implemented within the scope of ethics committee approval, may have resulted in the exclusion of certain patients whose perspectives could be relevant to the study findings. Therefore, the results may underrepresent the views of individuals with cognitive impairment or other conditions that hinder questionnaire completion. Furthermore, although the participants completed the questionnaire voluntarily, in some cases, the attending physicians may have chosen not to administer the questionnaire because the participant was judged as unable to respond accurately to the questions (e.g., due to suspected dementia). Given the voluntary nature of participation and the 44.5% response rate, individuals with cognitive impairment or lower health literacy may have been underrepresented, potentially limiting the generalizability of the findings and leading to overestimation of interest in home medical care. In addition, some patients who successfully completed the questionnaire may have had a cognitive impairment, such as dementia, which was unidentified by the physician. Moreover, the response rate was limited to 44.5%, suggesting potential bias toward respondents who were more willing to participate in the study. The study was limited to three clinics within the target region, necessitating caution when generalizing findings to other areas. While the findings provide valuable insights into rural healthcare dynamics, the study was limited to three city-operated clinics in a mountainous region of Sagamihara City. Therefore, the results may not be generalizable to urban settings or areas with different healthcare infrastructure or sociodemographic characteristics. Future studies should include diverse settings to better capture regional differences in attitudes toward home-based medical care. In the future, conducting longitudinal and comparative (comparisons with urban areas) studies as well as qualitative studies to gain a deeper understanding of residents' perspectives and attitudes that could not be captured in the present survey will provide more generalizable findings.

Interest in home-based medical care was assessed through self-reported data, which may not fully reflect actual intentions or behaviors. For example, when asking about “interest in home medical care,” it is important to recognize that patients may not be able to accurately predict their future preferences. Thus, self-reported interest should be interpreted as a proxy measure rather than a definitive indicator of future behavior, and future studies incorporating actual care utilization data would provide a more accurate assessment. This outcome measure may also be influenced by temporary circumstances, question framing, or social desirability bias and should be interpreted as a proxy measure rather than a definitive predictor of future utilization. Therefore, as a next step, we are considering conducting individual interviews to gather more practical data on the specific circumstances in which home-based medical care would be chosen as a preferred option, employing a qualitative research approach.

As this is a cross-sectional study, the observed associations do not necessarily imply causation. While certain associations were observed, these should be interpreted as hypothesis-generating rather than as evidence of causal relationships or policy effectiveness. The lack of statistically significant differences observed in some variables in this study may be attributable to data variability or limitations in sample size. In interpreting variables that were statistically significant, we reported descriptive numerical differences for completeness, but these should not be interpreted as indicative of real associations. Such differences may be due to sampling variability, and the study was not powered to detect small effect sizes in subgroup analyses. Future studies with larger sample sizes are needed to clarify these potential relationships. Practical constraints, such as economic factors and caregiver availability, were not comprehensively addressed.

Certain sociodemographic variables, such as educational attainment, household income, and access to information and communication technologies, were not collected because they were considered burdensome for respondents in this clinical survey context. The absence of these variables may have resulted in residual confounding, and future studies should include them to better clarify their impact on awareness and interest in home medical care. Although certain items were adapted from a municipal survey with demonstrated local relevance, the questionnaire was not pilot-tested or subjected to formal validation procedures. This may limit the reliability and generalizability of some measures, and future studies should incorporate validated instruments or perform pretesting to strengthen measurement robustness. Lastly, while interest in home-based care does not equate to a preference for home-based end-of-life care, this distinction warrants further investigation. In the logistic regression analysis, certain variables showed wide confidence intervals or p-values close to the significance threshold. These results may be less reliable, and the study was not powered to detect small-to-moderate associations with high precision.

Therefore, these findings should be regarded as exploratory. In addition, the regression models did not include several potentially important confounding variables, such as household income, educational level, and prior experience with home medical care, because these data were not collected. Therefore, residual confounding cannot be ruled out, and some of the observed associations may partly reflect these unmeasured factors. Future research should incorporate a wider range of sociodemographic and experiential variables to improve adjustment for confounding.

Strengths

Despite these limitations, the study's strengths lie in its systematic evaluation of factors influencing interest in home-based medical care based on responses from 807 participants across three clinics. The consistency of findings across clinics and demographic attributes reinforces the strong demand for home-based care services throughout the region.

## Conclusions

These findings suggest that factors such as older age, self-perceived poor health, and longer travel times to clinics were associated with greater interest in home medical care. However, given the absence of certain potentially important variables and the observed wide confidence intervals or p-values near the significance threshold for some estimates, these associations should be interpreted with caution. Instead, they may indicate priority areas for awareness-raising and service planning. Future longitudinal and intervention studies are warranted to confirm these relationships, assess their robustness, and evaluate their practical implications.

## Appendices

**Table 3:** Questionnaire items used in the survey.

Question no.	Item description	Response options
Q1	If you require long-term medical care due to illness, where would you prefer to spend your time? (Multiple answers allowed.)	1. At home. 2. In a hospital. 3. In a care facility. 4. Other (specify)
Q2	Do you know about home medical care?	1. Yes, I know about it. 2. No, I don’t know about it.
Q3	Do you think home medical care services should be improved in your area?	1. Yes, I think it’s necessary. 2. No, I don’t think it’s necessary. 3. I don’t know.
Q4	What is your current interest in home medical care?	1. I am not considering using it at this stage. 2. I might consider it depending on the conditions. 3. I am actively considering it. 4. I am not sure at the moment.
Q5	How would you describe your current health condition?	1. Healthy. 2. Somewhat healthy. 3. Somewhat unhealthy. 4. Unhealthy
Q6	How long does it take to get to the clinic from your home?	(______) minutes
Q7	Have you been hospitalized in the past year?	1. No. 2. Internal medicine. 3. Surgery. 4. Orthopedics. 5. Dermatology. 6. Ophthalmology. 7. Urology. 8. Otolaryngology. 9. Other (please specify: ________)
Q8	Do you have other regular hospitals or clinics you visit?	1. No. 2. Internal medicine. 3. Surgery. 4. Orthopedics. 5. Dermatology. 6. Ophthalmology. 7. Urology. 8. Otolaryngology. 9. Dialysis. 10. Dentistry. 11. Other (please specify: _______)
Q9	What is your household composition?	1. Living alone. 2. Living with a spouse (spouse aged 65 or older). 3. Living with a spouse (spouse aged under 65). 4. Living with parents (two generations). 5. Living with children (two generations). 6. Living with parents, children, and grandchildren (three generations). 7. Living near family (within the same premises or neighborhood). 8. Living in a shared facility. 9. Other (please specify: ________)
Q10	How did you get to the clinic today?	1. Train. 2. Bus. 3. Taxi. 4. Private car (driven by yourself). 5. Private car (driven by family or others).6. On foot. 7. Bicycle. 8. Motorcycle

**Table 4:** Missing data counts for each question and figure analysis. Valid n = number of respondents included in the analysis after listwise deletion of missing or "unsure" responses. Missing/unsure n (%) = number (percentage) of respondents excluded due to missing or "unsure" responses.

Figure	Variable	Valid n	Missing/unsure n (%)
Figure 1A	Preferences for long-term care locations	783	24 (3.0%)
Figure 1B	Knowledge of home medical care	795	12 (1.5%)
Figure 1C	Need for more home medical care in one’s own residential area	793	14 (1.7%)
Figure 2A	Age and interest in home medical care	559	248 (30.7%)
Figure 2B	Subjective health perspective and interest in home medical care	559	248 (30.7%)
Figure 2C	Time to hospital and interest in home medical care	555	252 (31.2%)
Figure 3A	Gender and interest in home medical care	559	248 (30.7%)
Figure 3B	Hospitalization in the past year and interest in home medical care	556	251 (31.1%)
Figure 3C	Concurrent visits to other hospitals and interest in home medical care	557	250 (31.0%)
Figure 3D	Household composition and interest in home medical care	555	252 (31.2%)
Figure 3E	Means of hospital visits and interest in home medical care	557	250 (31.0%)
